# Pulmonary Blood Volume Measured by Dual-Energy Computed Tomography Can Help Distinguish Patients With Pulmonary Hypertension

**DOI:** 10.3389/fcvm.2022.835655

**Published:** 2022-07-05

**Authors:** Kiara Rezaei-Kalantari, Kaveh Samimi, Hamid Zomorodian, Hooman Bakhshandeh, Maryam Jafari, Ali Mohammad Farahmand, Taleb Pourseyedian, Maedeh Sharifian, Salah Dine Qanadli

**Affiliations:** ^1^Rajaei Cardiovascular Medical and Research Center, Iran University of Medical Sciences, Tehran, Iran; ^2^Cardio-Oncology Research Center, Rajaei Cardiovascular Medical and Research Center, Iran University of Medical Sciences, Tehran, Iran; ^3^Hazrat Rasoul-e-Akram Hospital, Iran University of Medical Sciences, Tehran, Iran; ^4^Department of Radiology, School of Medicine, Iran University of Medical Sciences, Tehran, Iran; ^5^Department of Radiology, Ali Asghar Children Hospital, Iran University of Medical Sciences, Tehran, Iran; ^6^Department of Internal Medicine, Tehran University of Medical Sciences, Tehran, Iran; ^7^Cardiothoracic and Vascular Division, Department of Diagnostic and Interventional Radiology, Lausanne University Hospital and University of Lausanne, Lausanne, Switzerland

**Keywords:** pulmonary hypertension, pulmonary artery pressure, dual energy computed tomography pulmonary angiography, whole lung enhancement, pulmonary blood volume

## Abstract

**Purpose:**

To evaluate the correlation between whole lung enhancement (WLE) and pulmonary blood volume (PBV) obtained through dual energy computed tomography pulmonary angiography (DECTPA) and echocardiography-derived systolic pulmonary arterial pressure (SPAP).

**Methods:**

Sixty-eight patients who underwent DECTPA were enrolled in the study after giving informed consent. A transthoracic echocardiography was performed for all the subjects within 48 h of their DECTPA study to measure SPAP. The correlation of the two DECTPA-derived parameters, WLE and PBV, with SPAP was assessed. In addition, the predictive strength of these parameters was compared with that of traditional computed tomography (CT) signs of pulmonary hypertension (PH).

**Results:**

The SPAP value showed a moderate correlation with main pulmonary artery (MPA) diameter (*r* = 0.48, *P* < 0.001), while having a weak correlation with WLE (*r* = −0.33, *P* = 0.007), PBV (*r* = −0.31, *P* = 0.01) and MPA/ascending aorta (MPA/AA) ratio (*r* = 0.26, *P* = 0.03). On regression analysis, MPA diameter (B ± SE: 1.8 ± 0.6, *P* = 0.004) and WLE (B ± SE: −0.5 ± 0.3, *P* = 0.042) had significant association with SPAP. In addition, SPAP ≥30 mmHg was related to the right to left ventricular diameter (RV/LV) ratio [OR (CI 95%): 24.39 (1.3–573.2), *P* = 0.04] and reversely associated with PBV [OR (CI 95%): 0.96 (0.93–0.98), *P* = 0.005]. Acquired cutoff value of 83% for PBV showed sensitivity and specificity of 73% to identify SPAP ≥30 mmHg [AUC (CI 95%):0.727 (0.588–0.866), *P* = 0.008].

**Conclusions:**

Automated postprocessing calculation of iodine distribution analysis by DECTPA could be considered as an adjunctive tool to investigate for PH.

## Introduction

Pulmonary hypertension (PH) is a complex life-threatening condition affecting ~1 percent of the population, with increased prevalence in older age and patients with left heart failure (1, 2). Due to its non-specific clinical symptoms, the diagnosis might be delayed until advanced stages of the disease (3). While right heart catheterization (RHC) is the gold standard technique of confirming the diagnosis, it is usually primarily suggested by less invasive modalities such as doppler echocardiography or CT scans. Non-invasive modalities might also help reduce the need for repeat RHCs in the post- treatment follow up of these patients. Computed tomography pulmonary angiography (CTPA) is a pivotal part of diagnostic workup in patients suspicious for PH, providing detailed morphologic information on thoracic structures and pulmonary vasculature. The dual energy systems allow for synchronous image acquisition at different kilovoltages. These scans contribute to material differentiation, on the basis of three- material decomposition algorithms. In thoracic imaging, measurement of iodine distribution throughout the lung parenchyma represented as iodine maps has been used as an estimate of the pulmonary blood volume. The technique has the ability to add functional information to the mere morphological details yielded by conventional single energy systems and has shown good correlation with the corresponding pulmonary perfusion diagrams depicted by other perfusion studies (4–6). Recently, promising practical benefits of DECTPA have been shown by several studies, namely improving prognostication in acute pulmonary thromboembolism (PTE) (7) and enhancing diagnostic accuracy in chronic thromboembolic pulmonary hypertension (CTEPH) (8, 9). The correlation between absolute pulmonary artery pressure (PAP) value and DECTPA parameters has been the focus of a few recent studies with controversial results (9–12).

In this study, we evaluated the correlation of DECTPA parameters with echocardiography-derived SPAP, and compared the predictive power of these parameters with conventional CTPA signs of PH and right heart strain using a new patient-based image acquisition protocol.

## Method

### Study Population

**Figure 1:**
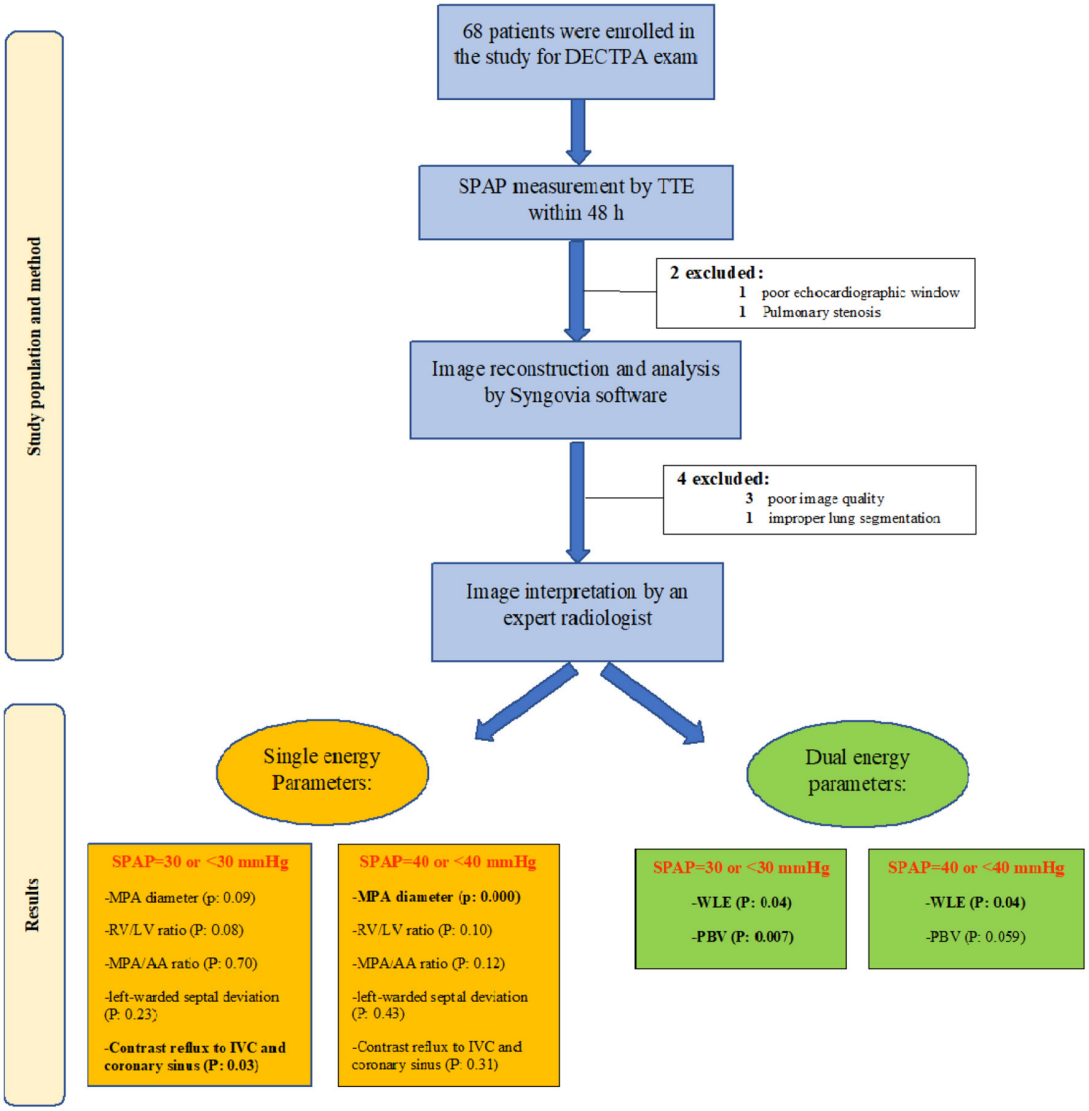
Study flow chart demonstrating the study population and method, as well as study results in groups of SPAP <30 and SPAP >30.

After study design approval from the research and ethics committee of our center, we enrolled 68 consecutive patients who underwent DECTPA between August 2019 and August 2020. All patients were given an informed written consent. A transthoracic echocardiography was performed within 48 h of their CT exam while no treatment was initiated for the patients within this period. Patients with pulmonary stenosis, very poor echocardiographic window, severe image artifacts, inadequate enhancement due to altered injection rate or improper lung segmentation by the software were excluded from the study (Figure 1).

### CT Protocol

All the DECTPA images were acquired on a dual-source CT scanner (SOMATOM Definition Flash; Siemens Healthcare, Forchheim, Germany) with the following scan parameters: tube voltage: Sn140 and 100 kVp; active CAREdose 4D; rotation time: 0.28 s; collimation: 12.8 × 0.6 mm; and pitch: 0.55. T. The pilot study with 10 patients revealed that the average volume CT dose index was 7.0 mGy/cm, with an average equivalent dose of 3.0 mSv, which was comparable to the conventional single source CTPA. An Iodine-based contrast material (Omnipaque 350; Daiichi-Sankyo, Tokyo, Japan) was chosen for all patients and injected through a 20-guage intravenous catheter into the right antecubital vein. All exams were preceded by a test bolus injection of 15 cc contrast material with a flow rate of 5.0 mL/s, followed by a 25-mL saline, to determine the MPA peak density time. The main dual energy exams were aimed to be acquired with total contrast dose of 0.5 ml/kg with the same injection protocol at the acquired time of peak MPA density.

### Image Reconstruction and Analysis

**Figure 2:**
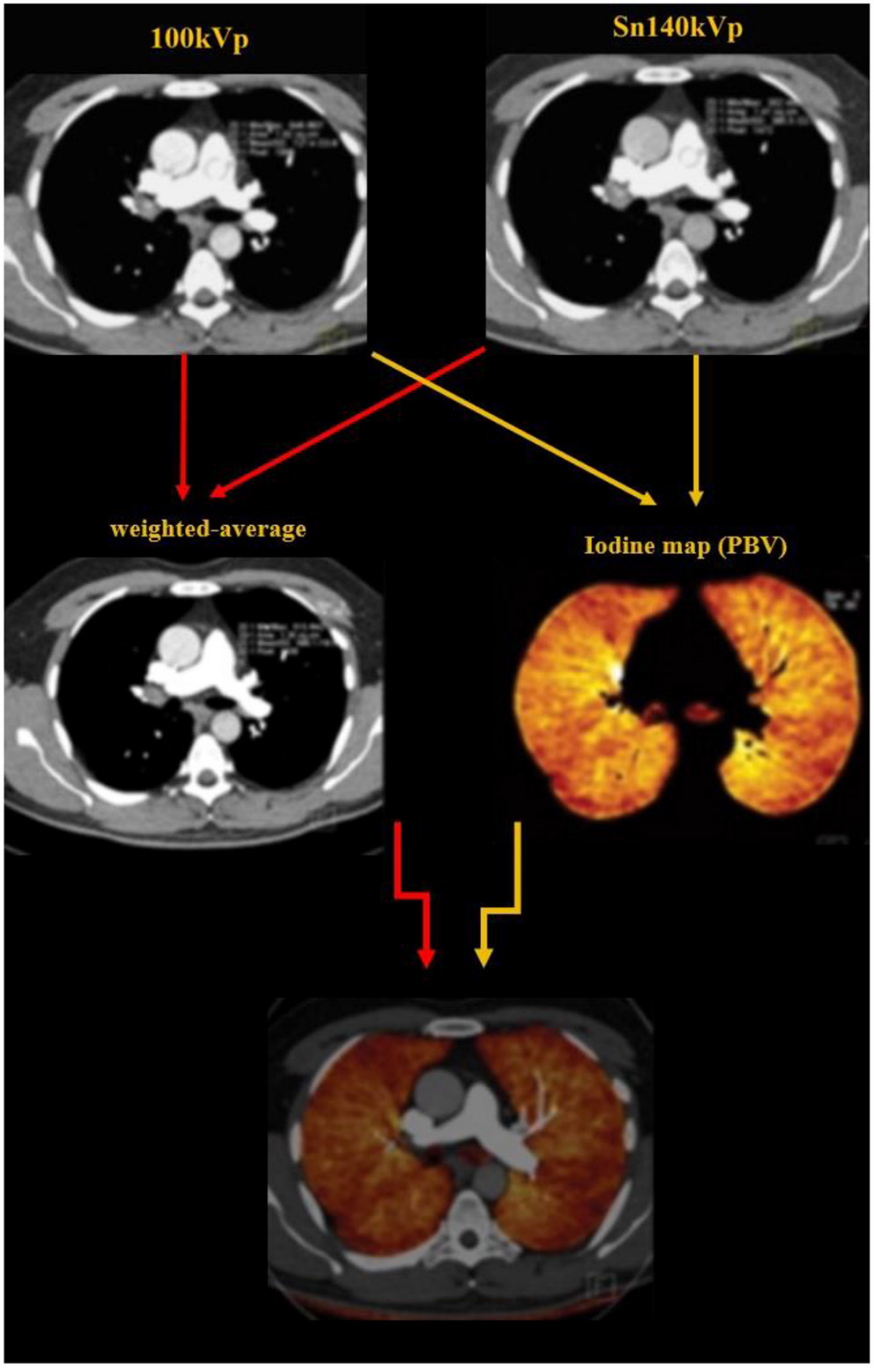
Image reconstruction was performed as three sets of images: 140-kV images, 100-kV images, and weighted-average images (with 60% and 40% weighting from Sn 100 kVp and Sn 140 kVp image series, respectively). The dual energy algorithm measures the attenuation in each voxel and uses the known standard attenuation ranges for the three major components of the lung parenchyma including the air, soft tissue and iodine, to calculate the relative contribution of each component to the voxel attenuation. The iodine distribution maps are subsequently created by the software.

Typical image reconstruction was performed as three sets of images: 140-kV images, 100-kV images, and weighted-average images (with 60% and 40% weighting from Sn 100 kVp and Sn 140 kVp image series, respectively) with a specific medium convolution kernel (D31) at a 2.0-mm slice thickness and a 1.0-mm increment (Figure 2).

**Figure 3:**
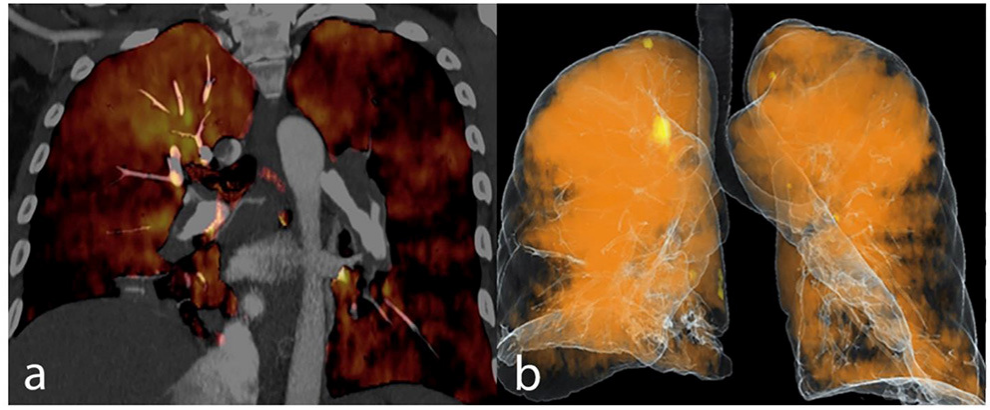
Dual energy CT pulmonary angiography in a CTEPH patient is demonstrated as iodine map overlay **(a)** and total blood volume image **(b)**.

Dual energy analyses were performed using the postprocessing software (Syngo MultiModality; Siemens Healthcare) with the “dual energy algorithm.” In this algorithm, the attenuation in each voxel is measure; using the known standard attenuation ranges for the three major components of the lung parenchyma including the air, soft tissue and iodine, the contribution of each component to the voxel attenuation is calculated. PBV maps were created from the images (Figures 2, 3) and whole lung enhancement (WLE) values were obtained. To measure the MPA enhancement, a region of interest circle of 10 mm^2^ was manually placed in the center of the arterial lumen on axial images, 1 cm before bifurcation. After normalizing the WLE based on MPA contrast enhancement, the PBV percentage (PBV) was automatically calculated by the software as an actual indicator of the total iodine distributed in the lung parenchyma relative to the iodine concentration in the MPA.

All the images were interpreted by one expert radiologist (with more than 10 years' experience in cardiothoracic imaging), blinded to the patients clinical and echocardiographic information. The maximum MPA diameter was measured in axial plane. The maximum AA diameter measurement was performed perpendicular to the long axis of the vessel. To calculate the RV/LV ratio, the widest short axis diameter of these chambers was measured from inner wall to inner wall, on reconstructed 4- chamber views. The presence or absence of septal bowing toward LV and reflux of the IV contrast into inferior vena cava (IVC) or coronary sinus was subjectively recognized by the radiologist.

### Echocardiography and SPAP Measurement

Transthoracic doppler echocardiography examinations were performed with Philips iE33 ultrasound system (Philips Medical Systems, Bothell, WA, USA) and a 5 MHz transducer, by two expert cardiologists with more than 10 years' experience in cardiology practice. The examinations were performed after at least 20 min of rest in supine position. The SPAP was measured by modified Bernoulli equation, using the tricuspid regurgitation velocity and the estimate of right atrial pressure based on the diameter and compliance of IVC. The SPAP ≥30 mmHg was defined as PH.

### Statistical Analysis

The statistical analysis was performed using IBM SPSS statistics 22 for windows (IBM Inc, Armonk, NY). The categorical variables were described as frequencies and percentages, and continuous variables were described as median and 25–75th percentile [interquartile ranges (IQR)]. The Spearman's correlation coefficient was used to investigate the correlation between SPAP and quantitative variables including age, PBV, WLE, MPA diameter, MPA/ AA ratio and RV/LV ratio. The Mann-Whitney U and Kruskal Wallis tests were utilized to evaluate the association between SPAP and categorical variables including sex, septal bowing and reflux of contrast material into IVC or coronary sinus. Multiple linear and multivariate logistic regression models were used to evaluate the association of conventional and dual energy parameters with SPAP. Thereafter, the accuracy of the mentioned parameters to determine SPAP ≥30 mmHg was determined by receiver operating curve (ROC) analysis. For all the tests, a *P*-value of <0.05 was considered as statistically significant.

## Results

### Baseline Characteristics

Of the 68 patients enrolled in the study, six were excluded due to pulmonary stenosis, poor echocardiographic window, poor image quality and improper lung segmentation by the software. Of 62 patients (median age of 53 years, females: 48.3%), 11 patients had acute and 13 patients had chronic PTE. The rest consisted of either normal or abnormal CT of other etiologies.

Baseline and cardiologic characteristics are summarized in Table 1.

**Table 1:** Baseline and cardiologic characteristics in total study group and subgroups according to the presence and type of PTE.

**Variable**		**All cases** ***N* = 62**	**No PTE** ***N* = 38**	**Acute PTE** ***N* = 11**	**Chronic PTE** ***N* = 13**
Age (years)		53 (35–62)	50 (34–61)	58 (39–67)	49 (33–60)
EF (%)		50 (40–50)	45 (35–55)	50 (40–50)	50 (45–50)
SPAP (mmHg)		40 (30–61)	39.5 (28–50)	35 (27–55)	61 (42–100)
WLE (HU)		31 (24–36)	32 (27–40)	27 (23–31)	26 (21–31)
PBV (%)		72 (53–95)	76.5 (54–98)	55 (39–110)	64 (50–85)
MPA diameter (mm)		29.8 (26.4–34.3)	28 (25–32)	30 (26–33)	34 (30–35)
MPA/AA		0.89 (0.76–1.04)	0.87 (0.75–0.97)	0.87 (0.75–1.03)	1.06 (0.92–1.25)
RV/LV		1.08 (0.91–1.24)	1.02 (0.84–1.15)	1.08 (0.88–1.24)	1.26 (1.06–1.32)
Sex	Female	30 (48%)	18 (47%)	6 (54%)	6 (46%)
	Male	32 (52%)	20 (53%)	5 (44%)	7 (54%)
reflux to IVC	No	33 (54%)	21 (56%)	5 (45%)	7 (54%)
	Yes	28 (46%)	37 (44%)	6 (55%)	6 (46%)
reflux to coronary sinus	No	26 (43%)	14 (38%)	4 (36%)	8 (62%)
	Yes	35 (57%)	23 (62%)	7 (64%)	5 (38%)
Leftward septal bowing	No	24 (40%)	16 (43%)	3 (27%)	5 (38%)
	Yes	37 (60%)	21 (57%)	8 (73%)	8 (62%)

*Values are median (Interquartile range) or n (%)*.

### Association Between CT Parameters and SPAP

The SPAP was associated with the presence or type of PTE (*P* = 0.024), as it was significantly higher in patients with chronic PTE than those without PTE or with acute PTE. The Qanadli score was comparable between patients with acute and chronic PTE (*P* > 0.5). The Spearman's correlation coefficient revealed that in the total study group, SPAP value had a moderate correlation with MPA diameter (*r* = 0.48, *P* < 0.001), and a weak correlation with WLE (*r* = −0.33, *P* = 0.007), PBV (*r* = −0.31, *P* = 0.01) and MPA/AA ratio (*r* = 0.26, *P* = 0.03). However, no significant correlation with age, gender, ejection fraction and RV/LV ratio, leftward septal bowing and reflux of contrast material into IVC or coronary sinus (*P* > 0.05 for all) were detected.

As shown in Table 2, both median WLE (*P* = 0.04) and PBV (*P* = 0.007) were significantly lower in PH patients compared to patients with normal SPAP. Also, the median WLE was significantly lower in thromboembolic patients compared to the other group of patients (26.5 HU vs. 32.5 HU, respectively, *P* = 0.007).

**Table 2:** Comparison of baseline and CT parameters according to SPAP of more or <30 mm Hg.

**Variable**		**All cases** ***N* = 62**	**SPAP <30** ***N* = 15**	**SPAP ≥30** ***N* = 47**	***P*-value**
Age (years)		53 (35–62)	58 (48–60)	49 (33–62)	0.35
EF (%)		50 (40–50)	50 (45–55)	45 (35–50)	0.07
WLE (HU)		31 (24–36)	35 (28–42)	29 (24–33)	**0.04**
PBV (%)		72 (53–95)	88 (72–104)	66 (49–88)	**0.007**
MPA diameter (mm)		29.8 (26.4–34.3)	28 (26–30)	31 (26–34)	0.09
MPA/AA		0.89 (0.76–1.04)	0.87 (0.81–0.98)	0.9 (0.75–1.05)	0.70
RV/LV		1.08 (0.91–1.24)	0.95 (0.83–1.14)	1.12 (0.92–1.28)	0.08
PTE type	No	38 (61%)	10 (67%)	28 (60%)	0.76
	Acute	11 (18%)	3 (20%)	8 (17%)	
	Chronic	13 (21%)	2 (13%)	11 (23%)	
Sex	Male	32 (52%)	8 (53%)	25 (52%)	0.93
	Female	30 (48%)	7 (47%)	23 (48%)	
Reflux to IVC	No	33 (54%)	11 (73%)	22 (48%)	0.13
	Yes	28 (46%)	4 (27%)	24 (52%)	
Leftward septal bowing	No	24 (39%)	8 (53%)	16 (35%)	0.23
	Yes	37 (61%)	7 (47%)	30 (65%)	
Reflux to Coronary sinus	No	26 (43%)	10 (67%)	16 (35%)	**0.03**
	Yes	35 (57%)	5 (33%)	30 (65%)	

*The significant P-values of less than 0.05 are shown in bold font*.

Table 3 demonstrates the CT parameters in two groups of patients with SPAP level of above and below 40 mmHg. Median WLE was significantly lower in SPAP ≥40 mmHg group [28 (23–33) vs. 31 (26–42), *P* = 0.04]. PBV also showed lower values in SPAP ≥40 group, albeit with borderline significance [64 (48–89) vs. 85 (64–98), *P* = 0.059].

**Table 3:** Comparison of baseline and CT parameters according to SPAP of more or <40 mm Hg.

**Variable**		**All cases** ***N* = 62**	**SPAP <40** ***N* = 28**	**SPAP ≥40** ***N* = 34**	***P*-value**
Age (years)		53 (35–62)	56 (37–62)	47 (32–61)	0.37
EF (%)		50 (40–50)	50 (45–55)	45 (35–50)	**0.03**
WLE (HU)		31 (24–36)	31 (26–42)	28 (23–33)	**0.04**
PBV (%)		72 (53–95)	85 (64–98)	64 (48–89)	0.059
MPA diameter (mm)		29.8 (26.4–34.3)	27 (23–30)	32.7 (29.4–35)	**0.000**
MPA/AA		0.89 (0.76–1.04)	0.86 (0.75–0.97)	0.93 (0.8–1.11)	0.12
RV/LV		1.08 (0.91–1.24)	1.03 (0.84–1.18)	1.14 (0.96–1.29)	0.10
PTE type	No	38 (61%)	19 (68%)	19 (56%)	0.21
	Acute	11 (18%)	6 (21%)	5 (15%)	
	Chronic	13 (21%)	3 (11%)	10 (30%)	
Sex	Male	32 (52%)	15 (54%)	18 (53%)	0.89
	Female	30 (48%)	13 (46%)	16 (47%)	
Reflux to IVC	No	33 (54%)	18 (64%)	15 (45%)	0.19
	Yes	28 (46%)	10 (36%)	18 (55%)	
Leftward septal bowing	No	24 (39%)	13 (46%)	11 (33%)	0.43
	Yes	37 (61%)	15 (54%)	22 (67%)	
Reflux to coronary sinus	No	26 (43%)	14 (50%)	12 (36%)	0.31
	Yes	35 (57%)	14 (50%)	21 (64%)	

*The significant P-values of less than 0.05 are shown in bold font*.

### Multivariate Analysis

For the assessment of the adjusted association between SPAP and CT indices, including the conventional and dual energy indices, a multiple linear regression model was applied and the following equation was obtained:

SPAP = 15.7^*^PTE−0.52^*^WLE+1.8^*^MPA diameter −8.7 (*R*^2^ = 0.35).

The model demonstrated that MPA diameter and chronic PTE had direct and WLE had reverse association with SPAP (*p*-values < 0.05). In this equation, PTE type is 0 for no PTE, 1 for acute PTE and 2 for chronic PTE; WLE is in Hounsfield unit, and MPA diameter is in mm.

Using a multivariate logistic regression model including CT parameters, we found that SPAP ≥30 mmHg was statistically associated with RV/LV ratio [OR (CI 95%): 24.39 (1.3–573.2), *P* = 0.04] and PBV [OR (CI 95%): 0.96 (0.93–0.98), *P* = 0.005]. According to ROC curves, a cutoff PBV value of 83% showed a good accuracy to identify patients with SPAP ≥30 mmHg with sensitivity and specificity of 73% [AUC (CI 95%):0.727 (0.588–0.866), *P* = 0.008].

Also, when considering 40 mmHg for discrimination, SPAP was directly associated with MPA diameter [OR (CI 95%): 1.31 (1.12–1.53), *P* = 0.001] and reversely associated with WLE [OR (CI 95%): 0.93 (0.86–0.99), = 0.049]. Based on the ROC curves, a cutoff value of 29.45 mm for MPA diameter showed a good accuracy to identify patients with SPAP ≥40 mmHg with sensitivity and specificity of 76 and 72%, respectively. [AUC (CI 95%): 0.796 (0.684–0.908), *P* = 0.000].

## Discussion

We sought to correlate the values of WLE and normalized PBV with echocardiography-derived SPAP. The distinguishing feature of this study that makes it prominent, is applying a test bolus method to find out the proper image acquisition time for each patient, and not a fixed delay time. This was done with the aim of precluding the confounding impact of cardiac function status and pulmonary hypertension level on the arrival of the contrast to the MPA and its distribution throughout the pulmonary parenchyma. Therefore, in each individual patient, the assessment was done at their peak MPA density and thus, the images and indices would be comparable between all.

According to our results, a statistically significant but weak negative correlation existed between both WLE and PBV with SPAP and on the regression model, WLE was a significant predictor of SPAP, as were for PTE type and MPA diameter. The weak to moderate correlation between PBV values and either mean or systolic PAP have also been shown by other investigators (9, 10, 12, 13). The correlation tended to be stronger in the studies which included CTEPH cases rather than normal controls or PH of other etiologies (10, 13, 14). Koike et al. demonstrated no significant correlation between PBV and echo-derived SPAP in their whole study group of patients with various PH etiologies. However, their subcategory analysis revealed that in some special PH groups including pulmonary artery stenosis, lung diseases and CTEPH, a weak to moderate correlation existed between PBV and SPAP. And interestingly, in patients with PH secondary to left heart failure, even a strong reverse (i.e., positive) correlation existed between PAP and PBV (11). In another study in CTEPH patients by Tsutsumi et al., such positive correlation was also demonstrated between PBV and mean PAP (14). The authors attributed their controversial results to the considered delay time for scanning—unlike early phase scans in other studies. And justified that the late phase images may actually reflect the confounding and compensatory systemic perfusion which reaches to the lungs in these patients. In our study, after excluding PH patients of heart failure etiology, no significant statistical change was detected in any of correlation indices; which might be related to the test bolus and not the fixed time protocol that we considered for assessment.

Our study results demonstrated that among both DECTPA and conventional parameters, the MPA diameter had the highest statistical correlation with SPAP, and was the major predictor of SPAP on regression models which is in accordance with published results by previous studies (9, 12, 15–17). A more recent study by Bacon et al. using “dual phase” DECTPA demonstrated that the change in WLE value between early and late phases was the only parameter to have a higher correlation with mean PAP than MPA diameter.

Our regression analysis revealed that PBV by DECTPA was a significant predictor of SPAP ≥30 mm Hg, and the cutoff of 83% had the sensitivity and specificity of 73% to identify individuals with mildly elevated SPAP. It is worth noting that the derived values for PBV and WLE vary according to the CT protocols and the normal ranges and diagnostic cutoffs should be individualized for each center.

In addition, RV/LV ratio was another notable predictor of SPAP ≥30 mmHg, although it did not have a significant direct correlation with SPAP values. This is also in concordance with previous published articles (9, 12, 15). One of them reported RV/LV ratio >1.28 as a significant predictor of PH with high sensitivity and specificity after controlling for cofounding factors of age, sex, pulmonary wedge pressure and indicators of body size (15).

In this study, MPA/AA ratio showed a weak association with SPAP. While the ratio has been shown by some to have an accuracy similar or even superior to MPA diameter (16, 17), a relatively weaker correlation has been reported by others (12, 14). The discrepancies might be due to diversity of study population (e.g., etiology, chronicity, ventricular function status) and different scan timing for measurement according to cardiac cycle.

DECTPA protocols are comparable to that of traditional single energy CTPA regarding the acquisition time, breath hold duration and the amount of administered contrast material (8). However, due to the dual energy nature of the exam, even a smaller amount of the contrast material could be injected for proper image acquisition (like in our study with the protocol of 0.5 ml/kg contrast injection) and the machine acquisition parameters (KV, mAs) could be adjusted to impose proportionate radiation dose to patient compared to conventional CTPA. Since the postprocessing calculation of PBV and WLE on DECTPA images is a feasible automated objective method, it might provide valuable incremental information, together with other traditionally known CT metrics, to investigate for pulmonary hypertension. DECTPA not only can help with posing the diagnosis of PH, but may also reveal the disease severity and help monitoring the treatment response.

### Limitations

The study might have been compromised by some limitations. First, the small sample size might have affected the results. Second, the SPAP in this study was estimated by echocardiography, which is not the gold standard modality for PAP quantification. Though, there is a good correlation between PAP measured by echocardiography and RHC in the literature (18).

## Conclusion

The automated post-processing calculation of WLE and PBV on DECTPA can act as a sensitive adjunctive tool to rise suspicion for PH in CT angiographies performed for various indications. Due to the insidious course and non-specific clinical picture of PH, dual energy parameters may help accelerate the diagnosis and improve the prognosis. Further larger studies are required to define the clinical applications of these parameters.

## Data Availability Statement

The raw data supporting the conclusions of this article will be made available by the authors, without undue reservation.

## Ethics Statement

The studies involving human participants were reviewed and approved by Rajaei Cardiovascular Medical and Research Center Ethics Committee. The patients/participants provided their written informed consent to participate in this study.

## Author Contributions

KR-K and KS contributed to conceptualization, methodology, and writing the original draft. HZ contributed to data acquisition, methodology, and reviewing the manuscript. HB contributed to methodology, statistical analysis, and reviewing the manuscript. MJ contributed to data acquisition, investigation, editing, and reviewing the manuscript. AF contributed to data acquisition, editing, and reviewing the manuscript. TP contributed to data acquisition and reviewing the manuscript. MS contributed to investigation, validation, and writing the original draft. SQ contributed to conceptualization, supervision, editing, and reviewing the manuscript. All authors contributed to the article and approved the submitted version.

## Conflict of Interest

The authors declare that the research was conducted in the absence of any commercial or financial relationships that could be construed as a potential conflict of interest.

## Publisher's Note

All claims expressed in this article are solely those of the authors and do not necessarily represent those of their affiliated organizations, or those of the publisher, the editors and the reviewers. Any product that may be evaluated in this article, or claim that may be made by its manufacturer, is not guaranteed or endorsed by the publisher.
